# Local Versus General Anesthesia in Pediatric Otoplasty: A Cost and Efficiency Analysis

**DOI:** 10.1177/10556656231186268

**Published:** 2023-07-02

**Authors:** Hari Iyer, Gabriel Bouhadana, Sabrina Cugno

**Affiliations:** 1 Division of Plastic and Reconstructive Surgery, 5622 Université de Montréal, Montreal, Quebec, Canada; 2 Division of Plastic and Reconstructive Surgery, 5620 McGill University, Montreal, Quebec, Canada

**Keywords:** esthetics, pediatrics, outcomes

## Abstract

**Objective:**

Quantify the cost benefits of otoplasty under local as opposed to general anaesthesia.

**Design:**

A cost analysis of all components of otoplasty surgery under local anaesthesia (LA) in a minor operating room (OR) and general anaesthesia in a main OR was performed.

**Setting:**

Our institution, compared to provincial/federal data, with costs converted into 2022 Canadian dollars.

**Patients, Participants:**

Patients undergoing otoplasty under LA in the last year.

**Interventions:**

An efficiency analysis was performed by means of an opportunity cost, and the cost of failure was added to the overall LA costs.

**Main Outcome Measure:**

Expenses for infrastructure, surgical and anaesthetic material, salaries, and personnel costs were derived from the literature, our hospital OR catalog and federal/provincial salary data, respectively. The cost of failure to tolerate local anaesthesia for such cases was also tabulated.

**Results:**

The true cost of LA otoplasty was computed as the absolute cost ($611.73) added to the cost of failure ($10.80), resulting in a total of $622.53/procedure. The true cost of GA otoplasty was calculated as the absolute cost ($2033.05) added to the opportunity cost ($1108.94), representing 3141.99$/procedure. The total savings when performing LA otoplasty to GA otoplasty are thus 2519.44$/case, with 1 GA otoplasty costing 5.05 LA otoplasties.

**Conclusion:**

Otoplasty under local anaesthesia offers significant cost savings when compared with the same procedure under general anaesthesia. Economic considerations must be given particular attention given the elective nature of this procedure, which is often publicly funded.

## Background

Prominauris remains the most common congenital external ear deformity, affecting around 5% of the population.^1^ Although essentially aesthetic in nature, it is often corrected in childhood given the potentially negative psychosocial impacts of this condition on children.^2^ The literature has focused heavily on the wide range of surgical techniques that have emerged for otoplasty,^3,4^ whereas less attention has been paid to the anaesthetic considerations and the economics of this procedure.

While the use of local anesthesia (LA) has been widely accepted for otoplasties among adults and its feasibility has been demonstrated in children,^5,6^ it has not been universally adopted in the pediatric population. Concerns mainly include patient discomfort/anxiety during injection, the possibility for post-operative negative psychological effects for both patients and their parents, and the potential for abortion of the surgery due to patient non-compliance.^5,6^ Nonetheless, our center has been performing otoplasties for children under LA since the early 1990's, presenting ways to successfully mitigate such concerns.^7^ Such a technique has yielded similarly low complication rates and high satisfaction rates when compared to general anesthesia (GA).^5,6,8^

Pediatric otoplasty in Canada is wholly reimbursed by the publicly funded healthcare system. In the context of increasing economic constraints, the lack of coverage for otoplasty by many private healthcare plans^9^ and the elective nature of the procedure, the impetus for minimizing associated costs has never been stronger. However, the specific cost differences between anesthetic techniques in the context of prominauris correction have yet to be formally studied. The goal of this paper is to therefore conduct a cost and efficiency analysis comparing local and general anesthesia for pediatric otoplasty, from a facility-cost perspective. The authors hypothesize that LA will carry important cost reductions, and hope this will contribute to a more universal acceptance of this technique among pediatric plastic surgeons.

## Materials and Methods

In our center, LA is considered the default option and GA is only considered under specific situations, including failure of patient compliance with LA. A review of all otoplasty patient charts from the last year was carried out to determine this failure rate. When performed under LA, the surgery is carried out in our minor operating room (OR), while GA is carried out in the main OR. Our specific LA technique has been previously described in detail.^7^

### Patient Selection

Children as young as 5 years of age may be considered candidates for otoplasty under LA. Foremost, a careful assessment of the child's intrinsic motivations should be conducted, as a child who is being coerced into ear surgery will likely not cooperate with the procedure under LA. Followingly, if surgery is requested, it is imperative to assess the child's ability to comprehend the surgery, explaining the procedure in simple terms. This is important, as getting the child to buy in to the surgical plan will strengthen their chances of complying with the LA. Finally, parental consent must be obtained, following a complete explanation of advantages and disadvantages of each anesthetic option. GA may be warranted if parents should want to proceed despite the child's incomplete understanding of the situation or due to their informed refusal of LA.

### Anesthetic Induction

Regarding local anesthesia, continuous conversation is carried out with the child throughout surgical site preparation and administration of anesthetic. This serves to distract the child from a foreign environment and unfamiliar, possibly painful sensations. The patient is first placed supine with their hands under their back. Following sterile preparation and draping, a total of 10cc of 1% lidocaine with 1:100,000 epinephrine is injected bilaterally using a 30-gauge needle. It is essential to make the child feel like they are in control, especially regarding taking a break from infiltration if pain is experienced. The posterior surface of the ear is first targeted, while the anterior surface is reached by passing the needle through the cartilage posteriorly.^7^ GA is carried out as per usual pediatric anesthetic protocols and preferences of the anesthetist.

### Surgical Technique

The senior author's (SC) preferred technique of otoplasty is a modified Mustardé conchoscaphal suture technique, but anterior scoring otoplasty is routinely carried out under LA at our institution and has been previously described in detail.^7,10^ Essentially, a posterior auricular skin incision is made overlying the lateral conchal bowl and the posterior aspect of the auricular cartilage is exposed in a subperichondrial plane. Conchoscaphal horizontal mattress sutures are placed to recreate the antihelix with nonresorbable material, and a stitch anchored to the mastoid fascia is placed to retroposition the concha if needed. A bolster dressing is placed after skin closure which remains intact for one week.

### Cost and Efficiency Analysis

Overall, the cost-analysis was carried out from an institutional perspective, and all values were converted to 2022 Canadian Dollars. Cost data were separated into three main categories: infrastructure, equipment/supplies and personnel. Only intraoperative costs were considered, thereby excluding pre- and postoperative care in the GA cost analysis. Infrastructure costs were derived from Steve et al.'s^11^ 2019 analysis of a minor OR in an academic Canadian hospital. Surgical and anaesthetic supply costs were obtained from our institution's specific OR catalog after review of our minor and major otoplasty surgical supply sheets. We included local anaesthetic supplies within surgical costs as this material was used in both procedures. Personnel costs were derived from provincial and federal salary databases: surgeon and anesthetist fees were obtained from the Régie de L’Assurance Maladie du Québec^12^ and nursing, respiratory therapist and orderly salaries from the Government of Canada Job Bank statistics.^13^ Failures were defined as a planned LA otoplasty that did not result in completion—usually due to child non-compliance. The basic cost of failure was defined as all LA costs barring surgeon fees (as no surgery is completed). The average cost of failure was calculated by multiplying this basic cost by the rate of failure and was added to the overall LA costs.

**Figure 1.**
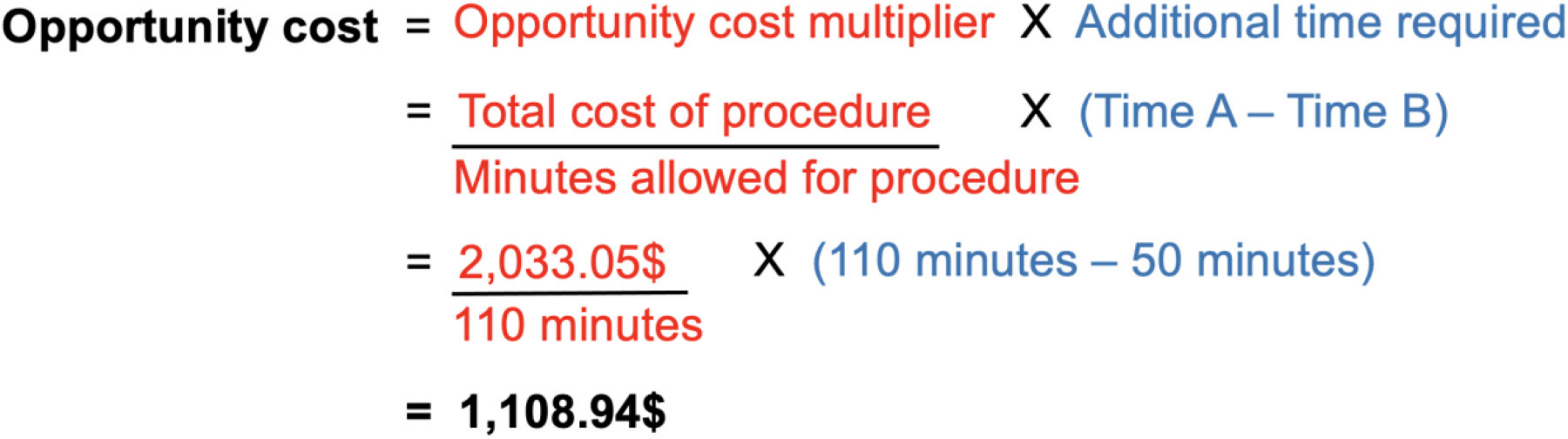
Calculation demonstration for opportunity cost.

For efficiency analysis, OR statistics were consulted to determine the mean in-room time for procedures under local and general anesthesia. This included time for general anaesthesia, injection of local anesthetic—performed in both procedures for hydrodissection, haemostasis and post-operative analgesia—while excluding turnover time. Based on these time differences, the opportunity cost was calculated, defined as the cost of time lost because of an inefficiency that could be used for other work,^14^ or the cost of extra time used in GA otoplasty which would otherwise permit a greater number of otoplasties if done under LA. This was added to the total cost to calculate the true cost of GA otoplasty as compared to LA (Figure 1).

## Results

### Cost Analysis

In terms of infrastructure costs, LA required an estimated 81.0$ while GA required 1064.0$,^11^ given they were respectively performed in minor and major ORs (Table 1). In terms of equipment/supply costs, LA required a total of 43.08$, while GA required a total of 168.71$ (Table 1). This difference was mainly due to anesthesia supplies. A more detailed description of individual items can be found in Supplementary Digital Content 1. In terms of personnel costs, LA required a total of 487.65$ while GA required 800.34$ (Table 1). This discrepancy was mainly due to the addition of anesthesia personnel and a scrub nurse.

**Table 1.** Summary of Costs Associated with Otoplasties Performed Under Local vs. General Anesthesia.

		Local Anesthesia	General Anesthesia
Infrastructure	Operating room overhead	$ 81.00	$ 1064.00
Equipment/ Supplies	Surgical Supplies	$ 43.08	$ 58.97
	Anesthesia supplies & medication	–	$ 109.74
Personnel	Surgeon fee	$ 457.57	$ 415.57
	Nurse fee	$ 30.08	$ 129.96
	Anesthetist fee	–	$ 158.40
	Respiratory therapist fee	–	$ 59.40
	Orderly salary	–	$ 37.01
Other	Cost of failure	$ 10.80	–
	Opportunity cost	–	$ 1108.94
TOTAL		$ 622.53	$ 3141.99

Based on our retrospective chart review, a total of 10 failures resulted from the 185 otoplasties performed under LA at our institution in the last year (5.4%). Followingly, the true cost of failure amortized over each successful case was equal the total LA cost without surgeon fees (160.61$) multiplied by the number of failures and divided by the annual number of successful cases (10/185-10), for a total of 10.80$.

### Efficiency Analysis

Based on our consultation of our institution's OR booking system, 50 and 110 min were allotted to LA and GA procedures, respectively. The opportunity cost multiplier was calculated as the cost of a GA otoplasty (2033.05$) divided by the time of a GA otoplasty (110 min), giving 18.48$/minute. This was multiplied by the additional 60 min it takes to complete an otoplasty under GA when compared to LA, which gives the opportunity cost per otoplasty case done under GA—yielding 1108.94$. In essence, this represents the cost of the extra time used in GA otoplasty, which could have been used for an LA otoplasty (Figure 1). Thus, the real GA cost can be represented as the absolute cost added to the opportunity cost, representing 3141.99$.

The total savings when performing LA otoplasty to GA otoplasty are thus 2519.44$/case (3141.99$–622.54$), when opportunity cost is factored in. In essence, this difference also represents the fact that, in terms of cost, 1 GA otoplasty could fund 5.05 LA otoplasties.

## Discussion

### Otoplasty Under Local Anesthesia is Economically Advantageous

Otoplasties remain one of the most common procedures performed by pediatric plastic surgeons. While many centers prefer to carry out this procedure under GA, our center has found success with LA. Although rarer, some recent studies describe the de facto use of LA in pediatric otoplasty surgery.^6,15,16^ This study demonstrates that there exists notable differences in terms of cost between otoplasties performed under LA vs. GA, especially when factoring in the associated opportunity cost. Our institution saves an estimated 2519.44$/case when performed under LA rather than GA, which represents a potential additional 4 otoplasties performed for the same overall cost. The time difference of 60 min represents added savings from a healthcare institutional perspective. Careful economic consideration must be given when planning otoplasty surgery, given its elective and aesthetic nature, and that it is often publicly funded or must be paid for out of pocket.

The overall economic advantage of LA otoplasty was likely underestimated by our study design. We purposefully included only intraoperative costs to allow direct comparison of LA and GA surgery, however this excludes significant institutional expenditure associated with GA. Nursing care and infrastructure of the day surgery unit where patients stay before and after surgery, as well as costs of post-anesthesia care unit (PACU) stay and potential post-operative hospitalization would all significantly increase the overall cost of GA otoplasty. Additionally, the parental economic burden of GA otoplasty is putatively higher, as these families spend the entire day in hospital whereas LA patients are free to leave after two hours at most, increasing flexibility around parents’ return to work and minimizing costs of childcare. Such expenses can have a significant impact on finances, especially for lower-income families.^17^ Eliminating the postsurgical effects of GA can subsequently accelerate return to school for patients and minimize absence from work for parents.^18,19^

### Otoplasty Under Local Anaesthesia Has Comparable Outcomes to General Anaesthesia

A previous studied published at our centre analyzing 500 otoplasty patients showed that local and general anaesthesia otoplasty differ in terms of age at surgery, but yield very similar outcomes.^8^ Our LA cohort had an average age of 8.8 years, while patients who had surgery under GA had an average age of 5.7 years. Acute complications were too rare in this cohort for stratification between anaesthetic techniques, with a 0.4% overall hematoma rate and 0.2% dehiscence rate over 500 cases. Late deformities, such as asymmetry and abnormal contour, were more common in the general anaesthesia group (16.4% vs 5.5%); this was attributed to a two-surgeon approach under GA and younger average age of these patients. Satisfaction rates based on post-operative follow-up notes were similarly very high (>94%) in both groups. These findings are largely homogeneous within the literature. Overall, patients who needed the surgery to be performed under GA are younger than patients in whom it is performed under LA.^5,6^ In addition, both yield similarly high satisfaction rates, with Hazkour and colleagues also reporting decreased postoperative parental anxiety and even slightly higher satisfaction after LA otoplasty.^6^ Finally, surgical complications are overall rare for this type of surgery, with no clear difference reported between anaesthetic techniques,^6,20^ and the need for revision is also uncommon.^5,6^

### Additional Advantages

Outside of cost, many other advantages exist to support LA otoplasty in children. LA avoids the overall risks of undergoing GA, and more practically allows patients to avoid post-operative nausea/vomiting (PONV)^5^ and to eat before the procedure.^18^ PONV is a common complication of general anesthesia, affecting up to 32% of pediatric patients according to a 2018 study,^21^ and can lead to hospital admission in rare cases. Additionally, avoidance of GA precludes the need for preoperative fasting while LA otoplasty has shown significant decreases in parental anxiety, increased willingness to repeat the procedure and lesser child trauma scores.^6^

The environmental impact of surgery at large is notable, with ever more single-use plastics being used for personal protective equipment, packaging, instrumentation and draping. Main OR surgical cases are associated with incrementally more such single-use plastics (Supplementary Digital Content 1). GA further contributes to environmental impact through use of disposable anesthetic materials and, most significantly, expulsion of hydrofluorocarbon anesthetic gases with significant global warming potential.^22^ One hour of anesthesia time has been demonstrated to be equivalent in terms of greenhouse gas emissions to between 18 and 470 miles driven in an average American car,^22^ depending on the specific gas used.

### Optimizing Otoplasty Under Local Anesthesia

Although many advantages to LA exist, the technique also carries some major potential drawbacks, hence why it is not universally accepted as the standard of care. Firstly, undergoing surgery without sedation has been posited to create discomfort and anxiety for the child and increased stress for parents.^5,6^ This may accentuate patients’ and their parents’ aversion to future medical/surgical procedures and to the healthcare system as a whole. Additionally, there always exists a risk of canceling the surgery due to patient non-compliance, which can be inconvenient for all parties involved.^5^ Nonetheless, many ways exist in order to optimize the implementation and success of LA for pediatric otoplasty surgery, which are summarized in Table 2.

**Table 2.** Recommendations to Optimize Otoplasty Under Local Anesthesia.

Category	Recommendation
Patient Factors	• Use simple/soft terms when explaining procedure (avoid frightening terms) - Better assessment of patient's understanding
	• Carefully assess patient's intrinsic motivation for procedure - Place child in front of mirror to show before/after
	• Assess potential ability to tolerate local anesthesia - Inform yourself regarding prior vaccines/dental clinic visits
Operative Environment	• Determine and address any anxiety-provoking concerns
	• Address the child with compassion
	• Play distracting background music/videos
	• Create positive reinforcement - Provide gifts (toys, stickers)
	• Avoid restraints
	• Avoid monitoring or IV equipment
	• Keep needles/surgical equipment out of child's sight
	• Keep full face exposed at all times
Anesthetic Technique	• Ensure excellent communication with child, especially regarding: - When they can expect pain - If they would like to pause
	• Consider application of topical anesthetic cream 60 min prior to the infiltration
	• Add bicarbonate to the anesthesic solution
	• Start with a 30 gauge needle
	• Pinch near the needle injection site to distract the child
	• Start on the posterior surface of the ear - Less pain since the skin is less adherent to cartilage
	• Inject while withdrawing
	• Take your time - Should take around 10–15 min - NOTE: Rapid soft tissue distension can be the most painful stimulus

#### Patient Factors

It is imperative to conduct a thorough preoperative assessment of the child's ability to remain calm and cooperative. Anecdotally, younger patients may be more reluctant at first.^5^ While age is a useful barometer, it cannot replace an assessment of the child's intrinsic motivation for surgery to change their auricular deformity, which in our experience is the chief determinant of intraoperative compliance. The patient's maturity level, motivations and comprehension of the procedure are factors that can concretize their motivation and lead them to accept, or reject, surgery. Intrinsic motivation can be effectively gauged through interview and by placing the child in front of a mirror and showing them the potential before/after.^6,7^ A thorough explanation of the procedure including its preoperative, intraoperative and postoperative steps is vital in improving patients’ understanding.^7^ Using a child-friendly vocabulary and avoiding frightening terms may help optimize the child's willingness to move forward with surgery. If any doubt exists, surgeons should keep in mind that the surgery can be delayed with no real consequences, and the patient re-evaluated at a later, more mature age.

#### Operative Environment

Immediately prior to and during the surgery, it is important to create a relaxed atmosphere. This begins even before the child is brought to the surgical suite, by determining and addressing the child's potential concerns,^23^ and by addressing them with compassion throughout.^5^ Inside the room, distracting background music/videos are often recommended.^5–7^ Our practice has had great success with constantly distracting the patient with conversation and asking questions of the child that demand some cognitive effort.^7^ Some authors suggest that positive reinforcement with rewards that are within eyesight can also be helpful.^6^ No monitoring or IV equipment is used, and all instruments including needles are hidden from the patient's view.^7^ Importantly, the patient's face is kept exposed at all times and restraints should not be used, but rather the patient told to lay supine on top of their hands to avoid reflexive movement.^6,7^ There is ongoing debate regarding the presence of parents during the surgery. Some authors advocate it may help in mitigating the child's anxiety, as parents can embrace their child and help in distraction.^5,6^ Others find it too difficult to juggle both the patients’ and their parents’ potential anxiety.^7^

#### Anesthetic Technique

Given injection of the anesthesia is the principle cause of pain/anxiety for the child, effort should be invested into optimizing this step. It cannot be understated that being honest with the child as to when they can expect discomfort is key,^6^ but even more importantly, to make them feel they are in control during the LA administration by taking breaks as they wish.^7^ In general, 1% lidocaine with epinephrine is used (a total of 10cc or less),^5–7^ and adding bicarbonate may help in decreasing pain by buffering the solution's acidity.^5,6^ The addition of Marcaine^16^ or Ropivacaine^24^ to the anesthetic solution for longer post-operative pain control has also been recommended. Although not used in our practice, the application of topical LA cream 60 min prior to the procedure can be a useful adjunct.^6,25^

In terms of the specific infiltration technique, direct injection into the auricle is preferable both for anesthesia and hemostasis.^6,7^ A 30-gauge needle is used while pinching near the needle injection site to distract the child. The posterior auricle is used as a starting point as its skin is less adherent to cartilage and thus minimizes pain of tissue distension.^7^ Slow infiltration is key, taking easily 10–15 min for both ears.^7^ Some authors propose a circumauricular block prior to infiltration of the pinna,^5^ while others argue for regional anesthesia rather than LA to reduce post-operative pain.^26^ Comparison has yielded equivocal results, thus simpler local infiltration should be employed.^27^

### Failure

Although needing to abort the surgery remains a potential risk, it is easily minimized through the ways discussed above. In addition, the literature demonstrates little to no risk of such. In a retrospective review of 41 cases of LA otoplasties, where the mean patient age was 11.9 years, Lancaster et al.^5^ reported 0 abandoned procedures. In a study of 13 patients with a younger mean age of 7.0, Hazkour et al.^6^ also showed 0 abandoned procedures. Finally, in their technical review including patients as young as 5, Laberge report <1% in terms of abandoned procedures. In our case, 10/185 cases resulted in failure, but this does not preclude future completion of these surgeries under LA. Regardless, our cost analysis has demonstrated that the cost associated with such is minimal, especially when compared with costs of GA.

### Limitations and Future Directions

The main limitation of our study is its generalizability. Given the lack of a similar cost analysis in the literature and of data availability, basing our costs on the literature and on our specific institution's costs allowed for a robust analysis, but may limit the generalization of its precise numbers. We recognize that undertaking surgery on an awake child carries a steep learning curve and established expertise in the specific procedure. Surgery under LA is also generally less optimal for resident training in the academic setting. Despite these limitations, we hope that more centers adopt this technique so as to optimize resource management while enacting further studies to determine the surgery's utility and conduct a true cost-effectiveness analysis.

## Conclusion

From an efficiency standpoint, 5.05 LA otoplasties can be performed for the cost of 1 GA otoplasty. This represents substantial financial savings, allowing for conservation and appropriate redistribution of precious hospital resources. The authors therefore believe LA should be favored over GA in the setting of pediatric otoplasty. This is especially true given the similar complication rates when performing otoplasty under LA, and that the surgery may be delayed until the child can properly tolerate LA with no true consequences. The adoption of such would generate important cost savings for our healthcare system, all while providing safer and equally effective patient care by eliminating the risks associated with GA. Nonetheless, LA should never be imposed, and the option for GA should remain on the table for patients and their parents.

## Supplemental Material

sj-docx-1-cpc-10.1177_10556656231186268 - Supplemental material for Local Versus General Anesthesia in Pediatric Otoplasty: A Cost and Efficiency Analysis Supplemental material, sj-docx-1-cpc-10.1177_10556656231186268 for Local Versus General Anesthesia in Pediatric Otoplasty: A Cost and Efficiency Analysis by Hari Iyer, Gabriel Bouhadana and Sabrina Cugno in The Cleft Palate Craniofacial Journal
