# Supplementary material for: Local Versus General Anesthesia in Pediatric Otoplasty: A Cost and Efficiency Analysis
Source: Cleft Palate Craniofac J. 2023 Jul 2;61(11):1879–85. doi: 10.1177/10556656231186268 (PMC11497746; doi:10.1177/10556656231186268)
Supplement: sj-docx-1-cpc-10.1177_10556656231186268 - Supplemental material for Local Versus General Anesthesia in Pediatric Otoplasty: A Cost and Efficiency Analysis [file sj-docx-1-cpc-10.1177_10556656231186268.docx]

**Supplemental Digital Content 1.** Detailed Equipment Costs

|  | Cost per item | Number required for Local Anesthesia | Number required for General Anesthesia |
| --- | --- | --- | --- |
| **Surgical Supplies** |  |  |  |
| Major basin drape set | $ 15.54 | - | 1 |
| Split sheet/draping | $ 5.86 | 1 | 1 |
| Surgeon gown | $ 2.87 | - | 1 |
| Mask | $ 0.30 | 1 | 1 |
| Surgeon hat | $ 0.10 | 1 | 1 |
| Nurses’ hat | $ 0.10 | 1 | 1 |
| Patient labels | $ 0.01 | 1 | 1 |
| Pharmacy labels | $ 0.01 | - | 1 |
| Needle board | $ 1.60 | - | 1 |
| Sterile marking pen | $ 2.63 | 1 | 1 |
| Suction tubing | $ 1.07 | - | 1 |
| Chlorhexidine skin prep sponges | $ 4.20 | 1 | 1 |
| Chlorhexidine scrub brush | $ 0.64 | 1 | 1 |
| 10-cc syringe | $ 0.09 | 1 | 1 |
| 18-g 1” Needle | $ 0.03 | 1 | 1 |
| 30-g 1/2” Needle | $ 0.05 | 1 | 1 |
| Sterile Gloves | $ 1.93 | 1 | 1 |
| #15 Blade | $ 0.09 | 1 | 1 |
| Suture × 1 (plain gut 5-0) | $ 4.89 | 1 | 1 |
| Fluff gauze | $ 0.60 | 1 | 1 |
| Nonadherent dressing (Jelonet) | $ 16.36 | 1 | 1 |
| Surgical supplies Total |  | $43.08 | $58.97 |
|  |  |  |  |
| **Anesthetic Supplies** |  |  |  |
| Gas Mask | $ 2.55 | - | 1 |
| Oropharyngeal airway | $ 1.29 | - | 1 |
| Gas Circuit | $ 7.10 | - | 1 |
| Anaesthetic Gas | $ 11.16 | - | 1 |
| CO2 Tubing | $ 1.24 | - | 1 |
| Glidescope blade | $ 50.00 | - | 1 |
| Filters (X2) | $ 3.12 | - | 1 |
| Endotracheal tube | $ 1.79 | - | 1 |
| Tape for ETT fixation | $ 1.11 | - | 1 |
| IV cannula | $ 0.70 | - | 1 |
| IV Dressing | $ 0.75 | - | 1 |
| IV tubing | $ 8.57 | - | 1 |
| Normal Saline IV Bag | $ 2.10 | - | 1 |
| Medications | $ 4.76 | - | 1 |
| Needles | $ 0.10 | - | 1 |
| Syringes (X6) | $ 2.00 | - | 1 |
| Suction & Tubing | $ 1.44 | - | 1 |
| Anesthetic supplies Total |  | 0$ | $99.78 |
|  |  |  |  |
| **Anesthetic Medication** |  |  |  |
| Propofol (1 x 200 mg vial) | $ 1.18 | - | 1 |
| Rocuronium (1/2 x 50 mg vial) | $ 1.02 | - | 1 |
| Fentanyl (1/2 x 100 mcg vial) | $ 0.30 | - | 1 |
| Dexmedetomidine (1/10 x 200 mcg vial) | $ 1.12 | - | 1 |
| Ondansetron (1 x 4 mg vial) | $ 0.92 | - | 1 |
| Dexamethasone (1/5 x 20 mg vial) | $ 0.22 | - | 1 |
| 1% Lidocaine/epinephrine  (1:100,000) (1/2 x 20cc vial) | $ 5.20 | 1 | 1 |
| Anesthetic Medication Total |  | $5.20 | $9.96 |
